# Multifocal invasive mucinous carcinoma of the breast

**DOI:** 10.1002/jmrs.379

**Published:** 2020-01-23

**Authors:** Melissa J. Ferguson

**Affiliations:** ^1^ BreastScreen ACT ACT Health Canberra City Australian Capital Territory Australia

**Keywords:** Breast sonography, invasive mucinous carcinoma

## Abstract

Mucinous carcinoma accounts for approximately 2% of all breast cancer and is a rare subtype of infiltrating ductal carcinoma. It often presents as a lobulated, well‐circumscribed mass on mammography, sonography, and magnetic resonance imaging and can therefore be mistaken for a benign lesion. This case report discusses a rare case of multifocal invasive mucinous carcinoma of the breast detected on screening mammogram. The histological attributes and various imaging findings of mucinous breast cancer are then discussed.

## Graphical abstract

Mucinous carcinoma accounts for approximately 2% of all breast cancer and is a rare subtype of infiltrating ductal carcinoma. This case report discusses a rare case of multifocal invasive mucinous carcinoma of the breast detected on screening mammogram.

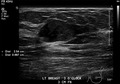

## Case History

A 75‐year‐old post‐menopausal female presented asymptomatic for routine screening mammography and was subsequently recalled to further assess multiple masses detected in her left breast upper outer quadrant. The patient has presented for routine biennial screening since 2015. The patient had no personal or familial history of breast or ovarian cancer. The patient reported the use of hormone replacement therapy for only a short period, with cessation fifteen years prior.

**Figure 1:**
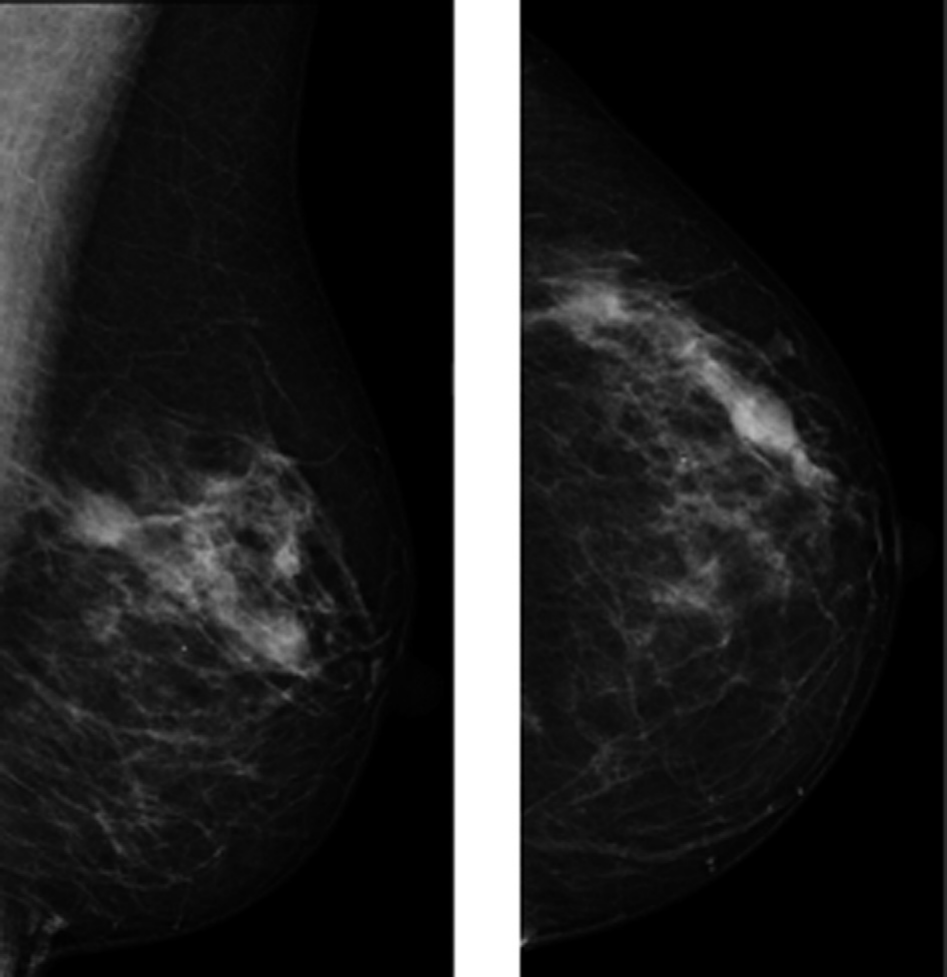
Mediolateral oblique (MLO) and craniocaudal (CC) projection showing multiple non‐specific densities in the left upper outer quadrant. Image courtesy of BreastScreen ACT.

**Figure 2:**
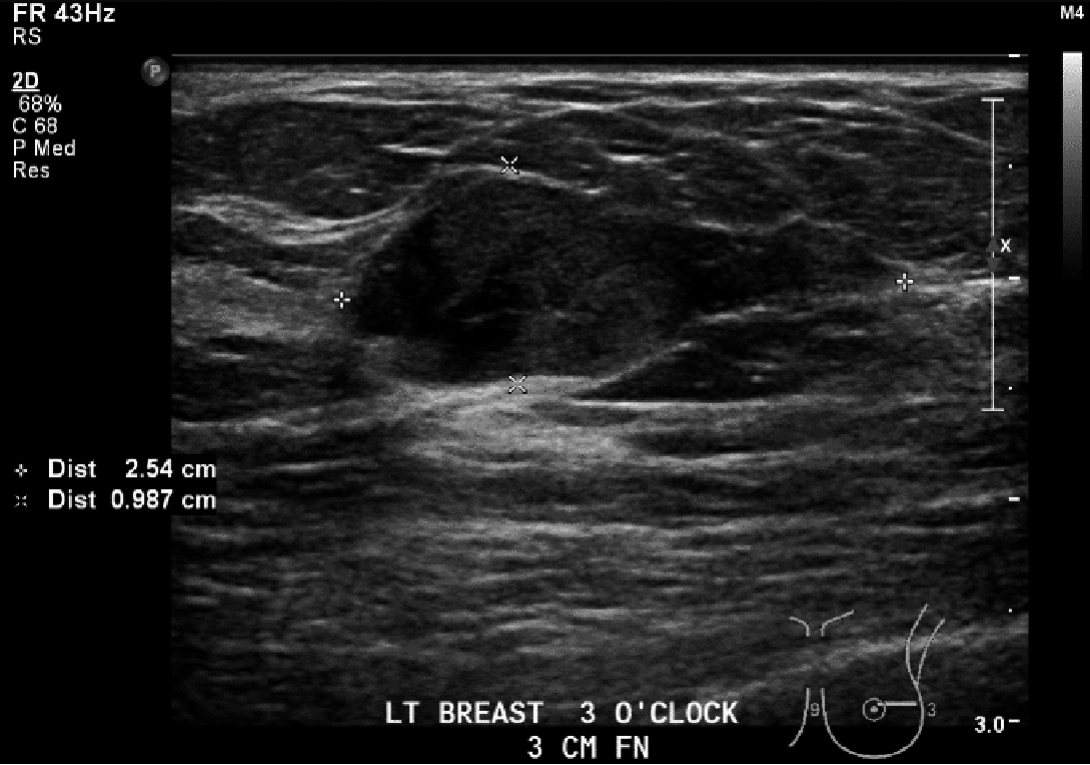
Lesion One: Ultrasound image showing a hypoechoic, well‐circumscribed mass with focus of internal calcification and small cystic component. Image courtesy of BreastScreen ACT.

**Figure 3:**
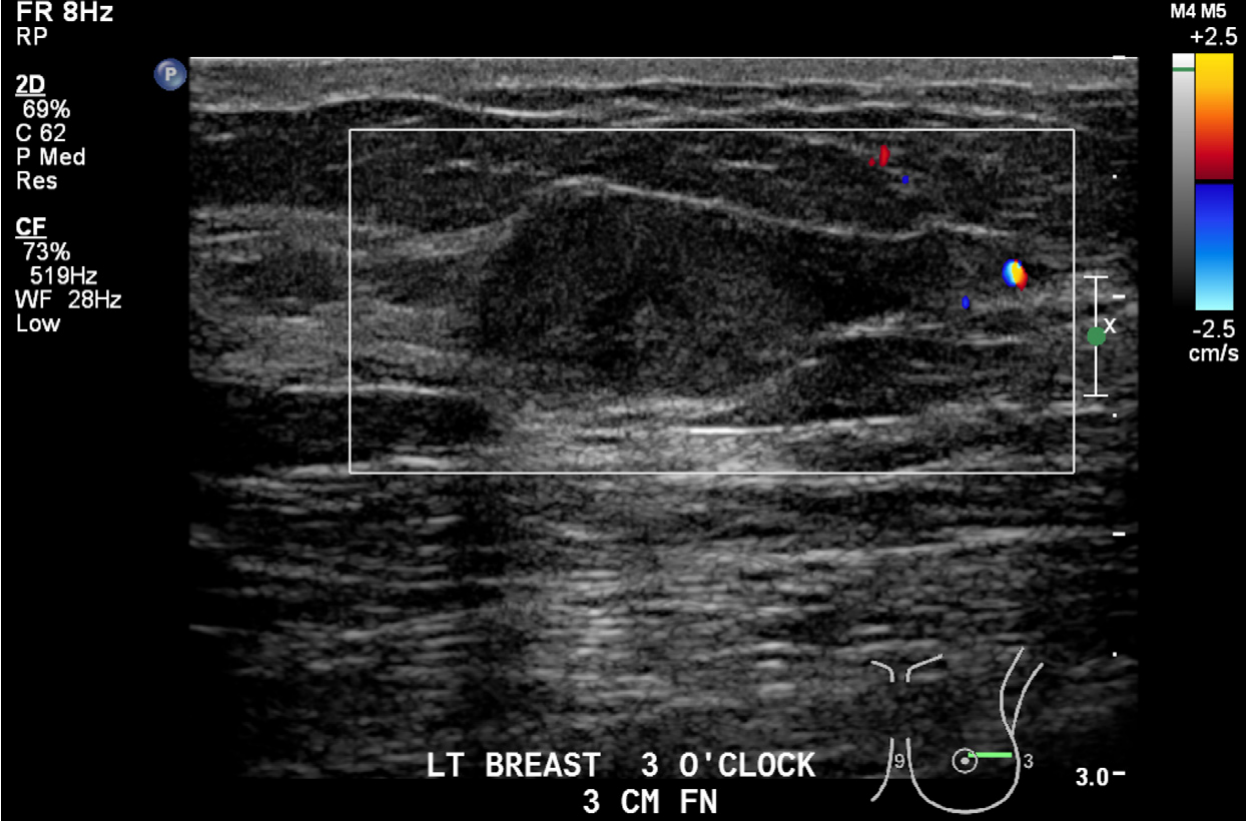
Lesion one with Doppler interrogation demonstrating mild vascularity. Image courtesy of BreastScreen ACT.

On returning for assessment, tomographic mediolateral oblique (MLO) and craniocaudal (CC) views were obtained and confirmed the presence of three, well‐circumscribed masses within the left breast upper outer quadrant (UOQ). Three, non‐tender lumps were palpated in the left breast UOQ on clinical examination which were thought to correspond with the masses detected on mammography (Fig. 1). Targeted ultrasound was subsequently performed and demonstrated three lesions as follows:

Lesion one (L1): Left breast 3 o‐clock, 3 cm FN; 25 × 10 × 15 mm hypoechoic, well‐circumscribed mass with focus of internal calcification and small cystic component (Fig. 2). Mild vascularity detected within the mass on Doppler interrogation (Fig. 3).

**Figure 4:**
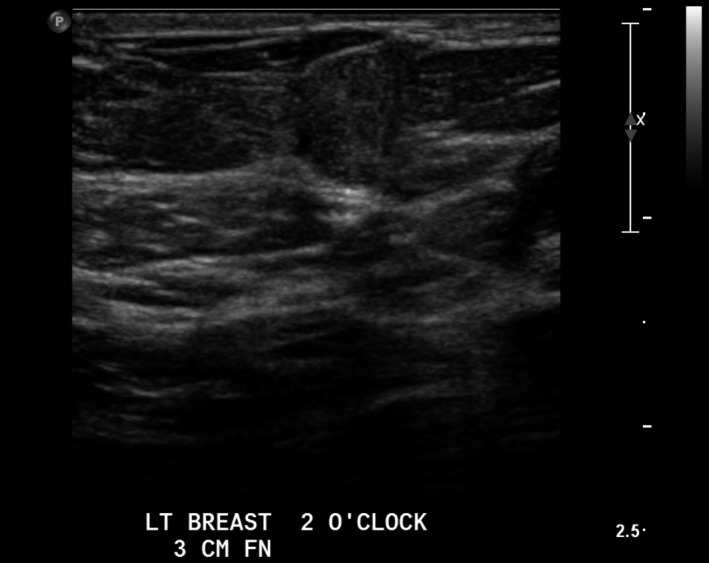
Lesion two: Ultrasound image showing a superficial, round mass adjacent to Lesion 1. Image courtesy of BreastScreen ACT.

**Figure 5:**
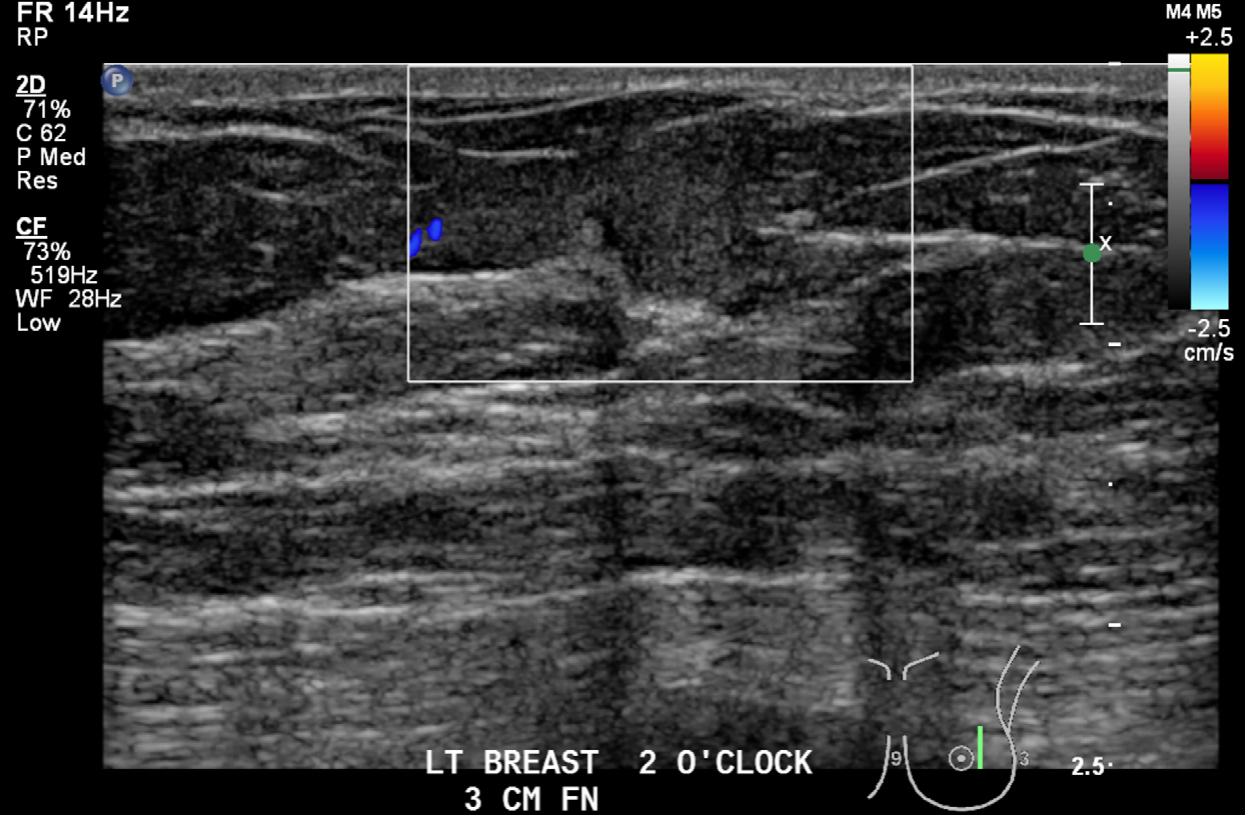
Lesion two with Doppler interrogation demonstrating avascular nature. Image courtesy of BreastScreen ACT.

Lesion two (L2): Left breast 2 o‐clock, 3 cm FN adjacent to lesion one; 9 × 8 × 6.5mm superficial, avascular, rounded, isoechoic mass with small cystic components. (Figs 4 and 5).

**Figure 6:**
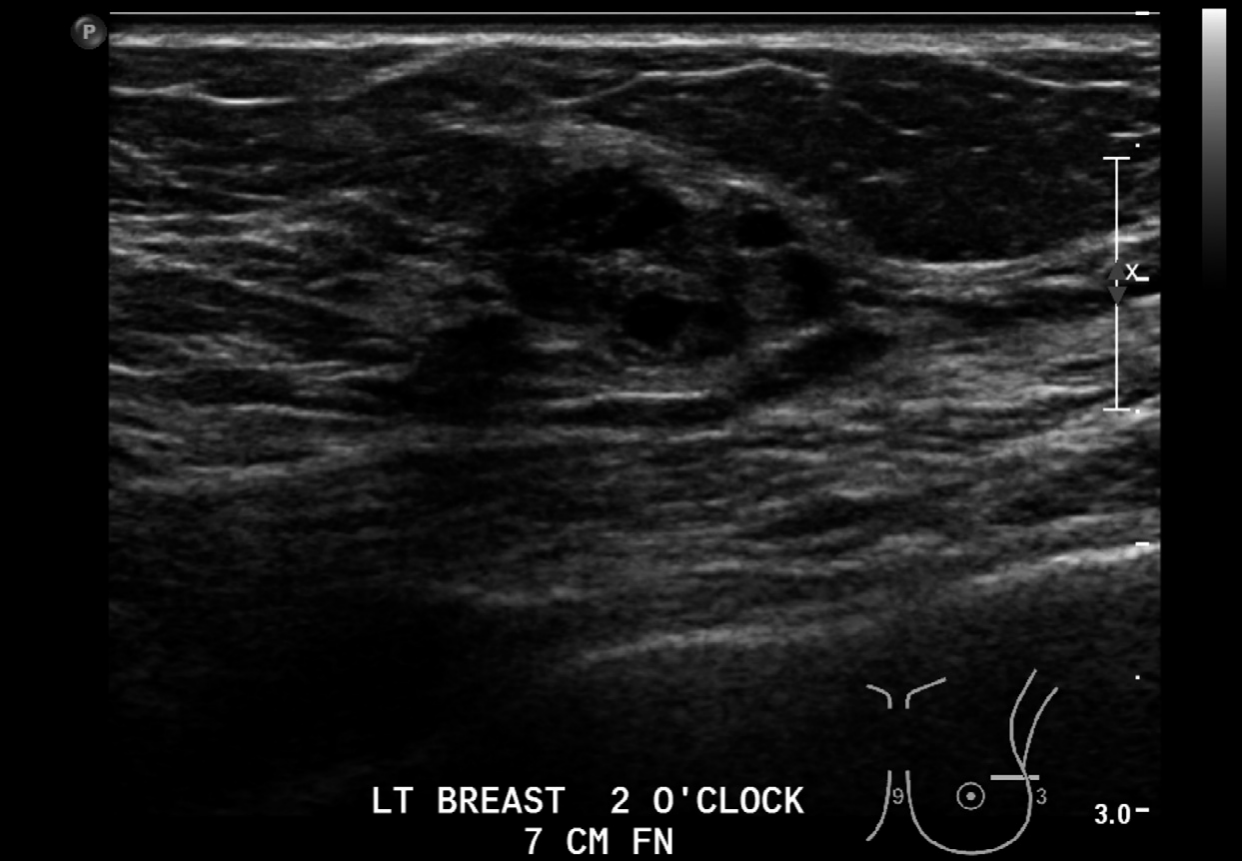
Lesion three: Ultrasound image showing an ovoid, mixed cystic and solid mass. Image courtesy of BreastScreen ACT.

**Figure 7:**
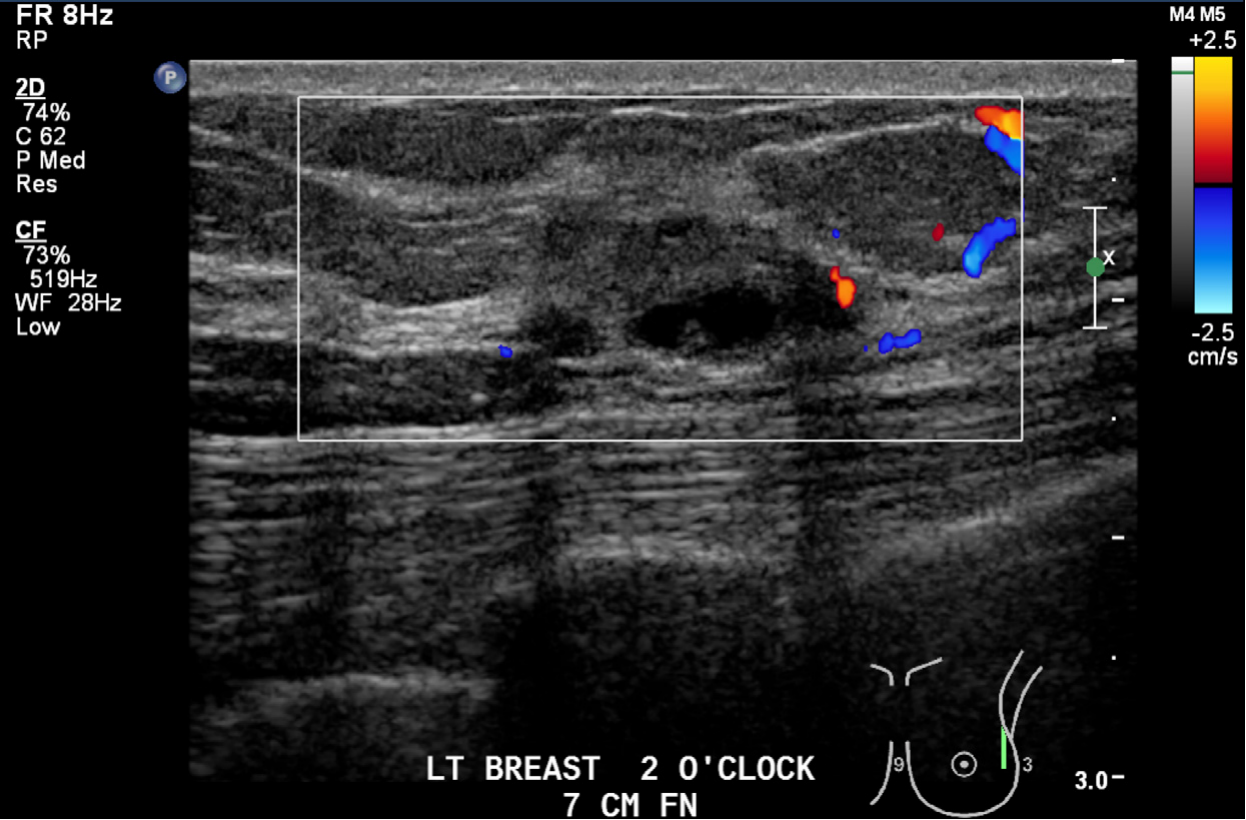
Lesion three with Doppler interrogation demonstrating avascular nature. Image courtesy of BreastScreen ACT.

Lesion three (L3): Left breast 2 o‐clock 7 cm FN; 14 × 8 × 17 mm, ovoid, mixed cystic solid, avascular mass with microlobulation (Figs 6 and 7).

### Provisional imaging diagnosis: papilloma (multiple) or papillomatosis

Ultrasound‐guided core biopsy of all three lesions was performed using a 14‐gauge biopsy needle and co‐axial technique. Three samples were obtained from each mass, and specimens were forwarded in formalin to pathology. The attending radiologist noted that the specimens obtained were friable and fragmented upon placing them in the specimen pots.

Histological analysis of the specimens from all three biopsy sites reported invasive mucinous carcinoma (mixed), predictive grade two. All three core biopsies show similar representative diagnoses with lesion three presenting areas of cribriform change.

Immunohistochemistry was performed, with positive staining for oestrogen and negative staining for progesterone and HER2. A ki‐67 index of 22 percent was reported.

### Definitive diagnosis: L1: invasive mucinous carcinoma; L2: invasive mucinous carcinoma; and L3 invasive mucinous carcinoma with cribriform area

A malignant diagnosis was established, and the patient has been referred for treatment and surgical intervention. Due to the widespread nature of the lesions within the breast, a total mastectomy was performed. During this, sentinel node testing was performed with two sentinel nodes removed, upon testing both were negative for malignancy.

Informed written consent was sought from the patient for publication.

## Discussion

Mucinous carcinoma is also referred to as colloid carcinoma, gelatinous carcinoma and mucous carcinoma.^1^, ^2^ It is an uncommon subtype of infiltrating ductal carcinoma and represents about 2% of all breast cancers. The incidence of mucinous carcinoma increases with age being approximately 1% in women < 35 years of age and 7% in women> 75 years of age.^3^ Additionally, mucinous breast carcinoma is extremely rare in men.^4^

Microscopically, mucinous carcinoma is characterised by a proliferation of clusters of generally small and uniform cells floating in large amounts of extracellular mucus, sometimes visible to the naked eye.^5^ As in this case, core samples obtained from mucinous carcinomas often fragment due to the presence of gelatinous material or mucin.

Pathologically, mucinous carcinomas are described as either pure or mixed. Distinction between pure and mixed lesions is important for prognosis and determining a treatment pathway.^3^ Pure mucinous lesions are defined by composition of at least 75% colloid pattern, 25% other components and consist of no non‐mucinous infiltrating ductal carcinoma, remaining virtually ‘pure’.^6^ Mixed lesions comprise of less than 75% colloid pattern and increased variety of mixed components.^3^ Mixed mucinous carcinomas are best managed as infiltrating ductal carcinoma not otherwise specified (NOS) due their mixed histological patterns.^5^

Pure mucinous carcinomas present on imaging as a well circumscribed, smooth, lobulated mass due to their greater mucin content. They are firm to soft on palpation depending on their mucin content.^3^, ^6^ A pure mucinous lesions resemblance to a benign process increases with increasing mucin content.^3^, ^5^

Mixed lesions typically have more infiltrating margins and are more likely to elicit a surrounding fibrous stromal reaction. This manifests as spiculated or ill‐defined margins on imaging. Due to the varied histological composition, mixed mucinous lesions tend to be firm to palpate on examination.^3^, ^6^

### Imaging

Mammographic imaging of pure mucinous carcinomas typically demonstrates a well circumscribed, smooth and lobulated density.^3^, ^6^ Mixed mucinous lesions may present with ill‐defined edges primarily owing to the presence of infiltrating reaction with surrounding fibroglandular tissue. Whilst uncommon, mammographic representations of mucinous carcinoma can demonstrate associated spiculation or calcification.

**Figure 8:**
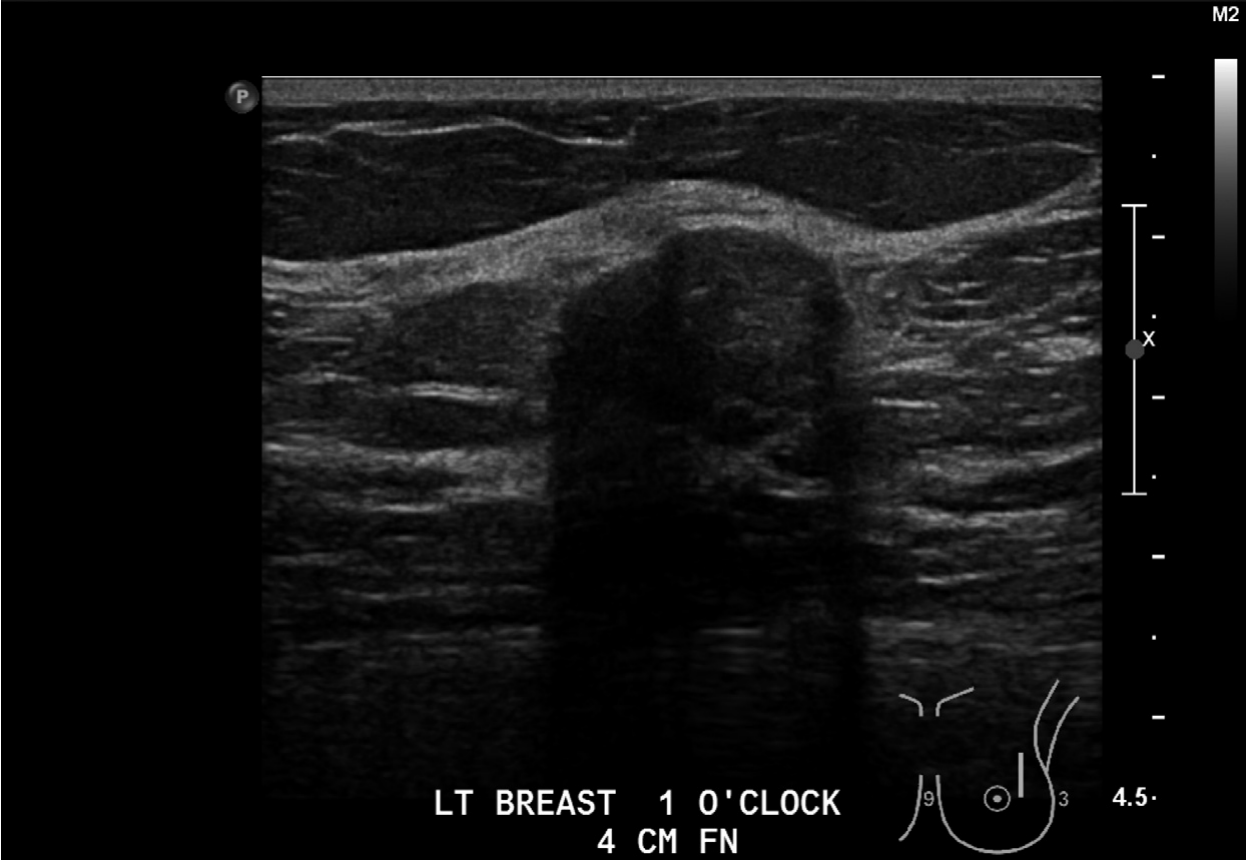
Ultrasound image demonstrating a fibroadenoma. Image courtesy of BreastScreen ACT.

**Figure 9:**
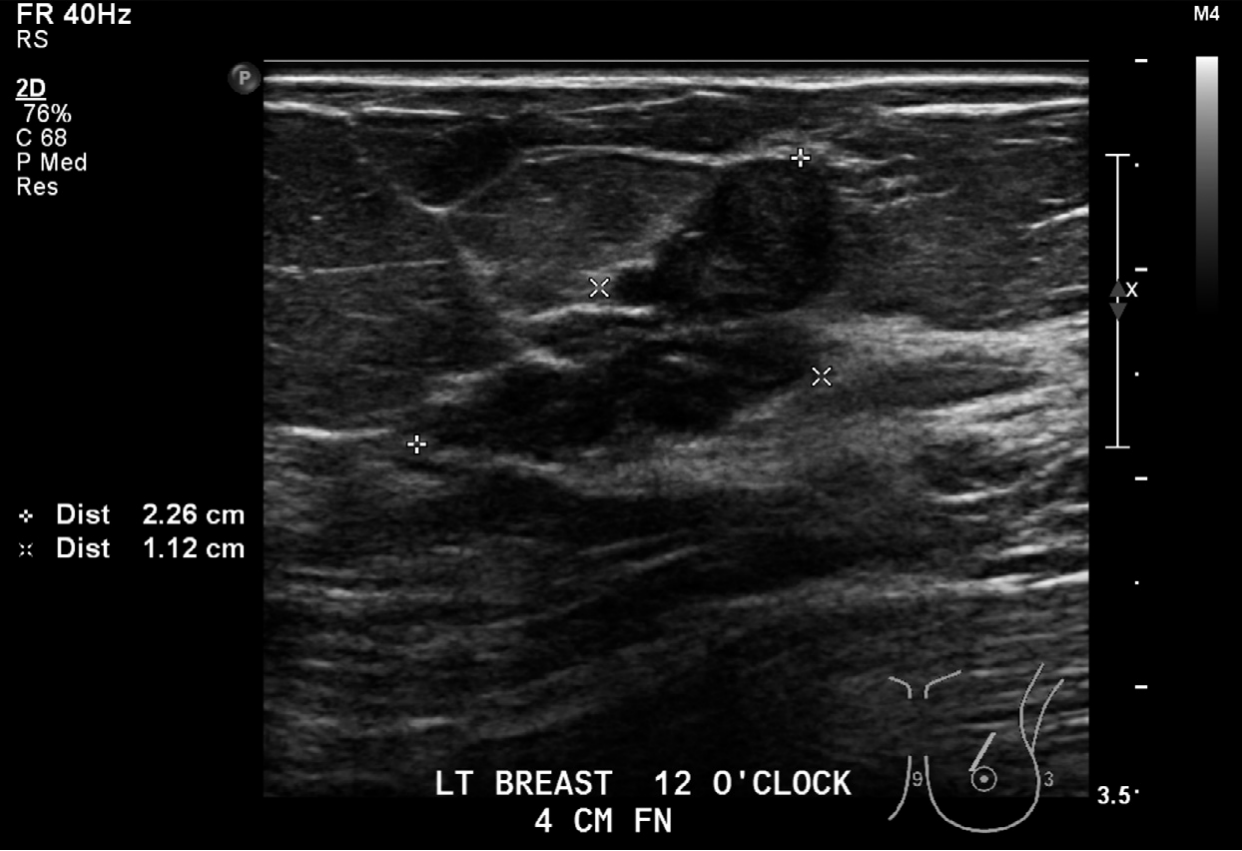
Ultrasound image demonstrating a known papillary lesion. Image courtesy of BreastScreen ACT.

Sonographic findings of mucinous carcinoma can have varied characteristics. Mucinous carcinomas typically present isoechoic, with a thin, well‐circumscribed echogenic capsule making them subtle to detection. Larger mucinous carcinomas can present with varied echogenicity, cystic components and potentially prominent duct extensions within the lesion. Mucinous carcinomas do not typically demonstrate posterior shadowing due to the enhanced through transmission. Due to the described nature of a mucinous carcinoma, it can be difficult to differentiate between benign processes such as normal fat lobules, lipomas, fibroadenomas (Fig. 8) or papillary lesions (Fig. 9), so additional imaging and biopsy should be discussed.^3^

Magnetic resonance imaging (MRI) typically demonstrates pure mucinous carcinoma as a relatively well‐circumscribed mass with a high fat‐saturated T2 signal and gradual rim‐enhancement. As previously discussed, the histological components of a mucinous carcinoma involve high amounts of extracellular mucin which is comparatively similar to benign findings on MRI. Features of contrast enhancement, signal intensity and reassessment of lesion margins can establish a differential diagnosis between a mucinous carcinoma and similar benign pathology (i.e. fibroadenoma). The use of MRI can also exclude the presence of any associated extensive intraductal components.^7^

### Multifocal nature of mucinous carcinoma

Mucinous carcinoma of the breast is considered a rare diagnosis,^2^ representing a very small portion of all invasive breast cancer types. In addition to this, the presentation of multifocal mucinous carcinoma of the breast is unique. The proliferative nature of a mucinous carcinoma is reported to be slow and non‐aggressive due to the nature of the mucin within the lesion surrounding the invasive tumour cells being involved.^1^, ^3^ Very few reference cases describe the diagnosis of multifocal mucinous breast carcinoma, making this case one of interest.

## Conclusion

The appearance of an invasive mucinous carcinoma of the breast can differ clinically, radiologically and pathologically. A comprehensive understanding of the varied nature a mucinous lesion is imperative to ensure consideration of appropriate interventional investigations are undertaken, in order to achieve a definitive diagnosis and facilitate sound future management.

## Conflict of Interest

The author declares no conflict of interest.
